# Supplementary material for: Effectiveness and Safety of Ayurvedic Medicines in Type 2 Diabetes Mellitus Management: A Systematic Review and Meta-Analysis
Source: Front Pharmacol. 2022 Jun 8;13:821810. doi: 10.3389/fphar.2022.821810 (PMC9213670; doi:10.3389/fphar.2022.821810)
Supplement: Supplementary file 1 [file DataSheet1.zip › Supplementary Material/Forest and Funnel Plots/Trigonella foenum-graecum (versus OAD)/FBG.pdf]

Trigonella foenum-graecum - FBG

	Intervention			Comparator			Mean Diff.	Weight
Study	N	Mean	SD	N	Mean	SD	with 95% CI	(%)
Singh 2016	20	124.30	14.93	20	113.70	12.99	10.60 [1.93, 19.27]	71.33
Najdi 2019	5	191.40	71.05	4	122.75	28.72	68.65 [-6.17, 143.47]	28.67
**Overall**							27.24 [-24.21, 78.69]	

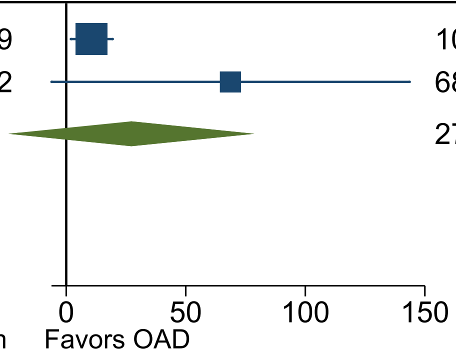

Heterogeneity: $\tau^2 = 946.53$, $I^2 = 56.18\%$, $H^2 = 2.28$

Test of $\theta_i = \theta_j$: Q(1) = 2.28, p = 0.13

Test of $\theta = 0$: z = 1.04, p = 0.30

Random-effects REML model
